# Association Between Atopic Dermatitis, Asthma, and Serum Lipids: A UK Biobank Based Observational Study and Mendelian Randomization Analysis

**DOI:** 10.3389/fmed.2022.810092

**Published:** 2022-02-21

**Authors:** Zhenwei Tang, Minxue Shen, Yi Xiao, Hong Liu, Xiang Chen

**Affiliations:** ^1^Department of Dermatology, Xiangya Hospital, Central South University, Changsha, China; ^2^Hunan Key Laboratory of Skin Cancer and Psoriasis, Changsha, China; ^3^Hunan Engineering Research Center of Skin Health and Disease, Central South University, Changsha, China; ^4^Department of Social Medicine and Health Management, Xiangya School of Public Health, Central South University, Changsha, China

**Keywords:** asthma, atopic dermatitis, serum lipids, UK biobank, Mendelian randomization

## Abstract

**Background:**

Both atopic diseases and dysregulation of serum lipids (SLs) add to significant health burden, but evidences about their association are inconsistent.

**Objective:**

This work is to evaluate the association between asthma/atopic dermatitis (AD) and SLs and investigate the potential causal relationship.

**Methods:**

A large-scale cross-sectional study based on the UK Biobank (UKB) and then examined the casual relationships between SLs with asthma/AD based on a Mendelian randomization (MR) analysis.

**Results:**

A total of 502,505 participants were included in analysis. After full adjustment, AD was associated with lower TG (β = −0.006; 95%CI, −0.010 to −0.002; *P* = 0.006), lower LDL (β = −0.004; 95%CI, −0.006 to −0.002, *P* < 0.001), and lower TC (β = −0.004; 95%CI, −0.005 to −0.002; *P* < 0.001) but insignificantly correlated to HDL (*P* = 0.794). Asthma was also inversely correlated to TG (β = −0.005; 95%CI, = −0.007 to −0.003; < 0.001), LDL (β = −0.003; 95%CI, −0.004 to −0.002; *P* < 0.001), and TC (β = −0.002; 95%CI, −0.003 to −0.002; *P* < 0.001), but was positively correlated to HDL (β = 0.004; 95%CI, 0.003 to 0.005; *P* < 0.001), respectively. In subsequent MR analysis, both allergic diseases and asthma showed a protective effect on TC. Allergic diseases, asthma, and AD all showed a negative effect on LDL.

**Conclusion:**

Collectively, we identify a protective causal effect of allergic diseases on serum lipids, as well as a potentially positive association of HDL with asthma. Owing to the largest sample size and the application of IVs in causal inference, this study will provide a robust evidence for the management of asthma and AD and the prevention of dyslipidemia.

## Introduction

Atopic diseases, including atopic dermatitis (AD), asthma and allergic rhinitis remain a great challenge worldwide. Allergic diseases affect ~10–30% population in developed countries and their global prevalence is still increasing (1, 2). Worse yet, the constant impact of atopic diseases often contributes to the onset of other comorbidities (3–5). To investigate the relationships between allergic diseases and their comorbidities are vital, since it may guide the treatment and management. Also, in some cases, observations on the atopic diseases and their comorbidities can lead to novel findings of the pathophysiology (4).

Serum lipids (SLs), including cholesterol, triglyceride, low density lipoprotein (LDL), high density lipoprotein (HDL), serve as an essential part of the energy supply of the whole body. On the other hand, the alteration of their concentration can lead to chronic vascular inflammation (6). Epidemiologically, unfavorable concentrations of SLs have been well clarified as a major risk factor for cardiovascular diseases (CVD), a leading cause of death worldwide. According to a previous estimation, over 50% global incidence of coronary artery disease (CAD) could be attributable to the dysregulation of SLs (7). More importantly, the association between dysregulated SLs and chronic diseases, such as psoriasis, psoriatic arthritis, diabetes, systemic lupus erythematosus, has been clarified apart from CVD (8–11). However, the association between atopic diseases and SLs remains greatly controversial. A meta-analysis of ten observational studies in 2017 suggested that the association between SLs and asthma was significant, but the effect varied from childhood to adulthood (12). On the other hand, a recent cohort study suggested that SLs might be associated with the diagnosis of asthma but not with its severity (13). More recently, an analysis of the cross-sectional data from the National Health and Nutrition Examination Survey (NHANES) in the U.S. revealed an insignificant association between SLs and asthma in children and adolescents (14). As for AD, only a few studies reported the potential association (15, 16). More evidences are needed to validate the casual relationship between atopic diseases and SLs and the underlying mechanisms.

Considering that previous inconsistent findings may be due to the sample size, age of the participants, and their incapability for causal inference, we herein conducted a large-scale cross-sectional study based on the UK Biobank (UKB) and then examined the casual relationships between SLs with asthma and AD based on a Mendelian randomization (MR) analysis.

## Methods

### Study Design and Participants

UKB is a large population-based study with over 500,000 participants, recruited during 2006 and 2010 from across England, Scotland, and Wales. The volunteers were aged between 40 and 70 at the time of recruitment, and provided data from touchscreen questionnaires, physical measurements, genotyping, and longitudinal follow-up, with further data continuing to be added (17). Because the SLs data was collected from all participants at baseline but was not repeatedly measured for most subjects during the follow-up period, we conducted a cross-sectional study rather than a longitudinal cohort study.

### Measurements and Definitions

Sociodemographic information, including age, sex, ethnicity, education, smoking status, alcohol consumption, household income, was obtained *via* face-to-face interviews or self-administered touchscreen questionnaires conducted at the baseline assessment center. Body height and weight were measured by research nurses, and body mass index (BMI) was then calculated. For asthma and AD, we used a variable that incorporated both self-reported history and medical diagnosis from inpatient or primary health care records.

Blood collection procedures are described in detail elsewhere and information on assay performance can be found on the UK Biobank website (18). SLs measured included total cholesterol (TC), LDL-C, HDL-C, and triglycerides (TG). Other biomarkers including fasting blood glucose (FBG), HbA1c, testosterone, and sex hormone-binding globulin (SHBG) were included as potential confounders. For the history of taking lipid-controlling agents (LCAs), we extracted related information based on the treatment/medication quires via verbal interview. Data fields of UKB used in this study were listed in Supplementary Table 1.

### Observational Study

We performed a cross-sectional analysis to test the association SLs of asthma/AD with SLs. First, concentrations of four SLs including TC, TG, LDL, and HDL were processed via testosterone logarithmic transformations owing to skewed distributions. Next, multivariable linear regression models were performed to estimate the associations of asthma/AD with different SLs. Based on previous studies about risks factors of SLs concentration (6, 19–21), we set the following models with adjustments for potential confounders: model 1 only included asthma and AD; model 2 further included age, sex, race, BMI, smoking status, alcohol intake, and household income in addition to model 1; and model 3 further included laboratory parameters including FBG, HbA1c, testosterone, and SHBG in addition to model 2. Since 4 parameters of SLs were involved, a *P*-value < (0.05/4) was considered statistically significant.

### Mendelian Randomization Analysis

We conducted a two-sample MR analysis based on a previous method (22). We used published summary statistic datasets from GWAS studies available on OpenGWAS database API (https://gwas.mrcieu.ac.uk/) (23), and only data from participants of European ancestry was included for the current analysis., we chose two datasets from the latest GWAS studies for TC, LDL, and HDL, and one dataset for TG. We chose one dataset for atopic diseases (including asthma, atopic dermatitis and hay fever), one for asthma, one for asthma with childhood/adulthood onset, and one for AD. Detailed information about the datasets we used is listed in Supplementary Table 2. Inverse-variance weighted (IVW) two-sample MR was performed using the R package “TwoSampleMR”, following the guidelines provided by the developers (https://mrcieu.github.io/TwoSampleMR), and in-house developed R scripts. Single nucleotide polymorphisms (SNPs) as instrumental variables (IVs) were carefully selected for association (*P* < 5 × 10^−8^) and processed for linkage disequilibrium (LD) removal (*r*^2^ > 0.05) via the clump data function in this package. The IVs used in this study were listed in Supplementary Table 3. Moreover, to test if this association was bi-directional, a reverse MR analysis, where the exposure and outcome were exchanged, was also conducted.

## Results

### Observational Study Based on UKB

A total of 502,505 participants were included in analysis. The sociodemographic information and related laboratory information are shown in Table 1. Generally, the mean age was 56.5 years, 52.7% were females, and 81.5% were Caucasian. Among the total participants, 13,822 participants were categorized as the AD group and 67,896 as the asthma group, with 3,071 participants overlapped in both groups. Most variables were significantly associated with AD/asthma (*P* < 0.05).

**Table 1:** Characteristics of participants by atopic dermatitis and asthma.

**Characteristics**	**Total (*N* = 5,02,505)**	**AD**		***P***	**Asthma**		***P***
		**No**	**Yes**		**No**	**Yes**	
		**(*N* = 4,88,683)**	**(*N* = 13,822)**		**(*N* = 4,34,609)**	**(*N* = 67,896)**	
Age (years), mean ± SD	56.53 ± 8.095	56.53 ± 8.097	56.53 ± 8.096	0.359	56.59 ± 8.069	56.16 ± 8.249	0.000
Female, *n* (%)	2,64,796 (52.7)	2,57,020 (52.6)	7,776 (56.3)	0.000	2,26,999 (52.2)	37,797 (55.7)	0.000
Body mass index (kg/m^2^), mean ± SD	27.43 ± 4.803	27.43 ± 4.799	27.56 ± 4.935	0.024	27.40 ± 4.690	28.29 ± 5.392	0.000
Caucasian, *n* (%)	4,09,615 (81.5)	3,98,237 (81.5)	11,378 (82.3)	0.014	3,54,487 (81.6)	55,128 (81.2)	0.021
Smoking, *n* (%)							
Never	2,73,522 (54.43)	2,66,204 (53.1)	7,318 (1.5)	0.004	2,37,327 (47.3)	36,195 (7.2)	0.000
Previous	173,056 (34.44)	168,083 (33.5)	4,973 (1.0)		148,859 (29.7)	24,197 (4.8)	
Current	52,978 (10.5)	51,530 (10.3)	1,448 (0.3)		45,903 (9.2)	7,075 (1.4)	
Alcohol, *n* (%)							
Never	22,385 (4.5)	21,760 (4.3)	625 (0.1)	0.125	19,013 (3.8)	3,372 (0.7)	0.000
Previous	18,104 (3.6)	17,569 (3.5)	535 (0.1)		14,892 (3.0)	3,212 (0.6)	
Current	460,362 (91.6)	447,743 (89.3)	12,619 (2.5)		399,270 (79.6)	61,092 (12.2)	
Household income, *n* (%)							
< 18,000	97,198 (19.3)	94,323 (19.0)	2,875 (0.6)	0.000	82,406 (16.6)	14,792 (3.0)	0.000
18,000–30,999	1,08,177 (21.5)	1,05,166 (21.2)	3,011 (0.6)		94,158 (19.0)	14,019 (2.8)	
31,000–51,999	1,10,772 (22.0)	1,07,786 (21.7)	2,986 (0.6)		96,585 (19.5)	14,187 (2.9)	
52,000–100,000	86,266 (17.2)	84,006 (16.9)	2,260 (0.5)		75,061 (15.1)	11,205 (2.3)	
>100,000	22,929 (4.6)	22,406 (4.5)	523 (0.1)		19,974 (4.0)	2,955 (0.6)	
FBG (mmol/L), mean ± SD	5.12 ± 1.243	5.13 ± 1.244	5.11 ± 1.239	0.220	5.12 ± 1.226	5.16 ± 1.352	0.849
HbA1c (mmol/mol), mean ± SD	36.13 ± 6.776	36.13 ± 6.784	36.10 ± 6.493	0.353	36.06 ± 6.696	36.60 ± 7.250	0.000
Testosterone (nmol/L), mean ± SD	6.56 ± 6.054	6.57 ± 6.056	6.18 ± 5.984	0.000	6.59 ± 6.065	6.31 ± 5.976	0.000
SHBG (nmol/L), mean ± SD	51.63 ± 27.781	51.60 ± 27.752	52.81 ± 28.789	0.000	51.80 ± 27.711	50.53 ± 28.200	0.000

First, we examined the association between SLs and asthma/AD by model 1 and found that serum concentrations of TC, TG and LDL were significantly associated with asthma and AD (*P* < 0.00125) (Table 2). In model 3 with full adjustment, AD was associated with lower TG (β = −0.006; 95%CI, −0.010 to −0.002; *P* = 0.006), lower LDL (β = −0.004; 95%CI, −0.006 to −0.002, *P* < 0.001), and lower TC (β = −0.004; 95%CI, −0.005 to −0.002; *P* < 0.001) but insignificantly correlated to HDL (*P* = 0.794) (Table 2). Asthma was also inversely correlated to TG (β = −0.005; 95%CI, = −0.007 to −0.003; *P* < 0.001), LDL (β = −0.003; 95%CI, −0.004 to −0.002; *P* < 0.001), and TC (β = −0.002; 95%CI, −0.003 to −0.002; *P* < 0.001), but was positively correlated to HDL (β = 0.004; 95%CI, 0.003–0.005; *P* < 0.001), respectively (Table 2).

**Table 2:** Associations of atopic dermatitis and asthma with serum lipids.

**Lipids**	**Model**	**Disease**	**Total**		**Lipid-controlling drug excluded**		**Caucasian only**	
			**β (95% CI)**	***P***	**β (95% CI)**	***P***	**β (95% CI)**	***P***
TC	Model 1	AD	−0.003 (−0.004 to −0.001)	1.33E-03	−0.002 (−0.004 to −0.001)	5.00E-03	−0.003 (−0.004 to −0.001)	1.94E-03
		Asthma	−0.002 (−0.003 to −0.002)	0.00E+00	−0.002 (−0.002 to −0.001)	1.41E-05	−0.002 (0.003 to −0.001)	5.77E-07
	Model 2	AD	−0.004 (−0.005 to −0.002)	0.00E+00	−0.003 (−0.005 to −0.002)	3.39E-05	−0.003 (−0.005 to −0.002)	4.44E-05
		Asthma	−0.002 (−0.003 to −0.001)	8.01E-08	−0.002 (−0.002 to −0.001)	2.71E-06	−0.002 (−0.003 to−0.001)	6.55E-06
	Model 3	AD	−0.004 (−0.005 to −0.002)	5.29E-05	−0.003 (−0.005 to −0.001)	1.00E-03	−0.004 (−0.006 to −0.002)	6.34E-05
		Asthma	−0.002 (−0.003 to −0.002)	7.98E-05	−0.002 (−0.003 to −0.001)	0.00E+00	−0.002 (−0.002 to −0.001)	1.38E-03
TG	Model 1	AD	−0.006 (−0.010 to −0.003)	1.00E-03	−0.005 (−0.010 to −0.001)	1.37E-02	−0.008 (−0.013 to −0.004)	1.46E-04
		Asthma	0.007 (0.006 to 0.009)	7.08E-15	0.007 (0.005 to 0.009)	1.17E-10	0.007 (0.005 to 0.009)	5.81E-11
	Model 2	AD	−0.005 (−0.009 to −0.002)	4.00E-03	−0.004 (−0.008 to −0.000)	3.00E-02	−0.007 (−0.011 to −0.003)	3.30E-04
		Asthma	−0.003 (−0.005 to −0.002)	0.00E+00	−0.003 (−0.005 to −0.001)	2.84E-03	−0.004 (−0.006 to −0.002)	3.38E-05
	Model 3	AD	−0.006 (−0.010 to −0.002)	6.00E-03	−0.004 (−0.009 to −0.000)	5.37E-02	−0.006 (−0.011 to −0.002)	4.25E-03
		Asthma	−0.005 (−0.007 to −0.003)	1.25E-07	−0.005 (−0.008 to −0.003)	8.38E-07	−0.006 (−0.008 to −0.004)	1.02E-07
LDL	Model 1	AD	−0.004 (−0.006 to −0.002)	0.00E+00	−0.003 (−0.005 to −0.001)	6.49E-04	−0.004 (−0.006 to −0.002)	5.71E-04
		Asthma	−0.004 (−0.004 to −0.003)	5.37E-14	−0.002 (−0.003 to −0.001)	4.83E-06	−0.003 (−0.004 to −0.002)	2.41E-10
	Model 2	AD	−0.004 (−0.006 to −0.002)	1.23E-05	−0.004 (−0.006 to −0.002)	4.23E-05	−0.004 (−0.006 to −0.002)	9.57E-05
		Asthma	−0.004 (−0.004 to −0.003)	2.00E-16	−0.003 (−0.004 to −0.002)	1.48E-12	−0.004 (−0.005 to −0.003)	5.75E-14
	Model 3	AD	−0.004 (−0.006 to −0.002)	0.00E+00	−0.004 (−0.006 to −0.001)	1.00E-03	−0.004 (−0.007 to −0.002)	2.31E-04
		Asthma	−0.003 (−0.004 to −0.002)	6.10E-10	−0.003 (−0.004 to −0.002)	8.86E-11	−0.003 (−0.004 to −0.002)	2.29E-07
HDL	Model 1	AD	0.003 (0.001 to 0.005)	8.00E-03	0.003 (0.001 to 0.005)	1.41E-02	0.003 (0.000 to 0.005)	2.35E-02
		Asthma	−0.001 (−0.002 to −0.000)	3.90E-02	−0.002 (−0.003 to −0.001)	5.69E-04	−0.001 (−0.002 to 0.000)	1.10E-01
	Model 2	AD	0.000 (−0.001 to 0.002)	5.82E-01	0.000 (−0.001 to 0.002)	7.42E-01	0.001 (−0.001 to 0.003)	4.82E-01
		Asthma	0.004 (0.003 to 0.005)	2.00E-16	0.003 (0.002 to 0.004)	6.54E-12	0.004 (0.003 to 0.005)	2.00E-16
	Model 3	AD	0.0002 (−0.002 to 0.002)	7.94E-01	0.000 (−0.002 to 0.002)	9.90E-01	0.000 (−0.002 to 0.002)	7.51E-01
		Asthma	0.004 (0.003 to 0.005)	2.00E-16	0.003 (0.002 to 0.004)	9.90E-09	0.004 (0.003 to 0.005)	8.68E-16

In sensitivity analysis, we checked whether the association was modified by the administration of LCAs or genetic background. We extracted the participants without a history of taking LCAs and those of Caucasian origin, respectively (Table 1). The associations SLs retained significant with minor alterations in effect size among those reporting no history of taking LCAs (Table 2).

### Mendelian Randomization

**Figure 1:**
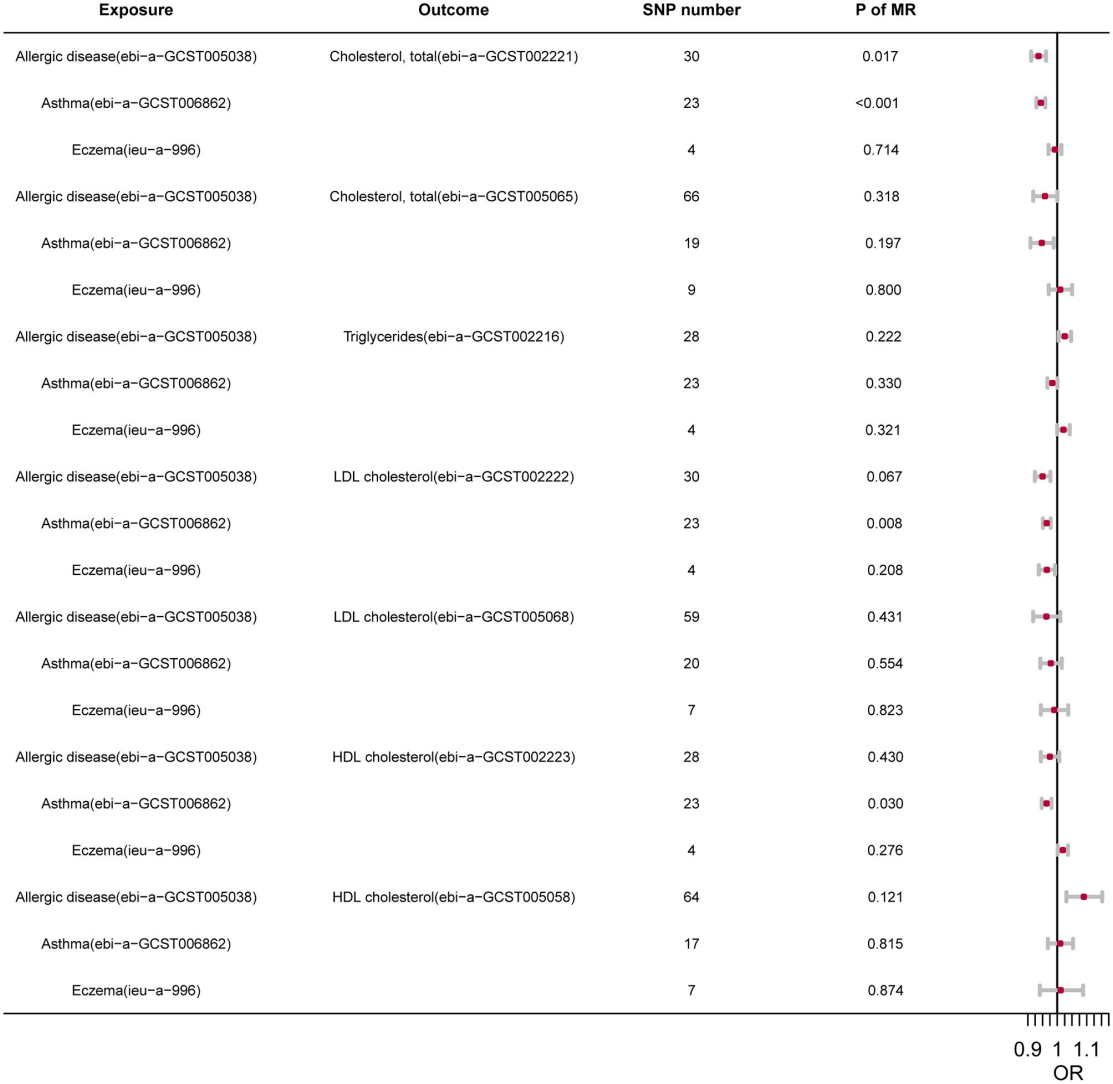
The summary of MR.

In order to illustrate the potential causal relationship, we further conducted two-sample MR analyses. After removal of LD and harmonization, different numbers of IVs were included in the final analysis (Figure 1). Despite that the associations were not statistically significant in some datasets, both allergic diseases and asthma showed a protective effect on TC. In contrast, the association of TG with atopic diseases was not significant (*P* > 0.05). Allergic diseases, asthma, and AD all showed a negative effect on LDL. The effect on HDL, however, exhibited a high inconsistency among different outcomes.

To test the horizontal pleiotropy of our analysis, we conducted the MR-Egger regression of each MR pair, and the results indicated no significant pleiotropy (Supplementary Tables 4–7). Besides, to reveal whether SLs could have a causal effect of asthma/AD, the reverse MR analysis was also performed, and no significance was found (Supplementary Tables 4–7).

## Discussion

To our knowledge, this study was based on the largest sample size and the first MR analysis to describe the relationships between SLs and asthma/AD. We found that both asthma/AD were negatively associated with TC, TG, and LDL. Subsequent MR analysis revealed a genetical casual effect of asthma on TC and LDL.

So far, both atopic diseases and SLs dysregulation have raised public concern. Evidences about their association, however, are highly heterogenous. A meta-analysis in 2017 suggested that HDL was significantly lower in asthmatic children and LDL was significantly higher in asthmatic adults (12). But the evidences included in this meta-analysis seemed highly heterogenous. Even based on the same data resource, results can be inconsistent. For instance, Fessler et al. and Lu et al. investigated the association between SLs and asthma using data from NHANES from 2005 to 2006 and from 1999 to 2012, respectively (14, 24). The former study revealed that TC and non-HDL-C are inversely correlated to asthma. The latter, however, claimed that there was no significant association between SLs and asthma, suggesting a need for expanded study. Besides, atopic diseases usually occur early during childhood, while the dysregulation of SLs are often observed in middle aged or even later. Majority of previous studies were based on younger populations such as children or adolescence, which are not considered to be representative for dyslipidemia research. In this study, we identified a negative association between TC/LDL and asthma/AD using a mid-aged population. The result was inconsistent with the previous studies, and we suspected that this was resulted from the differences in the age of participants and sample size (12–14).

Concerning the casual relationships between SLs and atopic diseases, most evidences were from cross-sectional studies and cannot indicate a causal relationship. Due to the beneficial role of calorie-restriction on atopic diseases, more attention was paid on investigating whether SLs dysregulation can contribute to the onset of atopic diseases (12, 16, 25–27), which may leave a bias on investigating the casual relationships between SLs and atopic diseases. Therefore, we explored the causal relationships through MR analyses, which showed consistent results with the observational study, supporting the protective effect of asthma/AD on lowering TC/LDL. The reverse MR further indicated that the casual effect of asthma/AD on SLs was unidirectional, indicating a non-existence of casual effect for SLs on allergic diseases. Taken together, our findings challenged the previous concepts that atopic diseases might contribute to dyslipidemia and on the contrary, proposed that atopic diseases might be intrinsically protective for dyslipidemia.

Our study also shed light on basic research. Inspired by the findings from MR analysis, we suspected that the lowering of SLs concentration might attributed to the genetic background of allergic population. Variations in human leukocyte antigen region (HLA) have been well-clarified to be associated with allergic diseases by numerous GWAS studies (28). One small-scaled study proposed an inverse correlation between HLA-DR expression and serum triglycerides concentrations (29). Results from another observational study indicated that variation in HLA-DQB1 were positively associated with lipid homeostasis and human longevity (30). Unfortunately, these studies merely revealed the association but did not provided further functional evidence, and future effort should be taken on the mechanical role of allergy-associated variation in dyslipidemia and CVD. On the other hand, allergic diseases are often featured or by an enhancement of Th2 cell-mediated responses. As the major and direct consequence of dyslipidemia, atherosclerosis can be attenuated by Th2-associated cytokines, such as IL-5 and IL-13, according to previous studies (31). Although the debate remains, studies also suggested that a higher proportion of Th2 cells among peripheral blood lymphocytes is positively correlated with lower subclinical atherosclerosis burden, and IL-4, another critical cytokine related to allergy, also inversely correlates with clinical atherosclerosis (31–33). Whether the protective role of Th2-associated cytokines is mediated by lipid metabolism, remain to be explored in the future.

Collectively, we identify a protective causal effect of allergic diseases on serum lipids. Owing to the largest sample size and the application of IVs in causal inference, this study will provide a robust evidence for the management of asthma and AD and the prevention of dyslipidemia.

## Data Availability Statement

Publicly available datasets were analyzed in this study. This data can be found in the article/supplementary material. Further inquiries can be directed to the corresponding author/s.

## Ethics Statement

This study was based on UKB (Application No.55257), and all individuals in this cohort provided written informed consent. North West Multi-Centre Research Ethics Committee approved the UK Biobank ethical application. The patients/participants provided their written informed consent to participate in this study.

## Author Contributions

ZT drafted the manuscript. MS and ZT analyzed the data. MS and YX designed the study. MS, HL, and XC obtained the funding. All authors participated in the design, data collection, critically revised the manuscript, and gave final approval to the version submitted for publication.

## Funding

This work was supported by the National Key Research and Development Project of China Precision Medicine Initiative (#2016YFC0900802) and the Program of Introducing Talents of Discipline to Universities (111 Project, #B20017). The funders did not participate in this study.

## Conflict of Interest

The authors declare that the research was conducted in the absence of any commercial or financial relationships that could be construed as a potential conflict of interest.

## Publisher's Note

All claims expressed in this article are solely those of the authors and do not necessarily represent those of their affiliated organizations, or those of the publisher, the editors and the reviewers. Any product that may be evaluated in this article, or claim that may be made by its manufacturer, is not guaranteed or endorsed by the publisher.
