# Supplementary material for: Association Between Atopic Dermatitis, Asthma, and Serum Lipids: A UK Biobank Based Observational Study and Mendelian Randomization Analysis
Source: Front Med (Lausanne). 2022 Feb 21;9:810092. doi: 10.3389/fmed.2022.810092 (PMC8899503; doi:10.3389/fmed.2022.810092)
Supplement: Supplementary file 1 [file Data_Sheet_1.doc]

**SUPPLEMENTARY FILE CAPTIONS**

**Table S1. UKB Code used for data extraction.**

**Table S2. Details of datasheet used in this study.**

**Table S3. IVs' information**

**Table S4. MR of TC**

**Table S5. MR of TG**

**Table S6. MR of LDL**

**Table S7. MR of HDL**

**Table S1. UKB Code used for data extraction.**

| Information | Field ID |
| --- | --- |
| Sex | 22001 |
| Age | 21022 |
| Race | 22006 |
| BMI | 21001 |
| Smoking | 20116 |
| Alcohol | 20117 |
| Household income | 738 |
| TC | 30690 |
| TG | 30870 |
| LDL | 30760 |
| HDL | 30780 |
| FBG | 30740 |
| HbA1c | 30750 |
| testosterone | 30850 |
| SHBG | 30830 |
| Lipid-controlling agents | 20003 |
| Atopic dermatitis | 131721 |
| Asthma | 131495 |

**Table S2. Details of datasheet used in this study.**

|  | API ID | Phenotype | Samplesize | Cases/Control | PMID |
| --- | --- | --- | --- | --- | --- |
| Ferreira et al(2017) | ebi-a-GCST005038 | Allergic disease (asthma, hay fever or eczema) | 360838 | 180129/180729 | 29083406 |
| Demenais et al(2017) | ebi-a-GCST006862 | Asthma | 142486 | 23948/118538 | 29273806 |
| Paternoster et al(2015) | ieu-a-996 | Atopic dermatitis | 377550 | 53458/324092 | 26482879 |
| Willer et al(2013) | ebi-a-GCST002221 | Cholesterol, total | 188577 | N/A | 24097068 |
| Prins et al(2017) | ebi-a-GCST005065 | Cholesterol, total | 9961 | N/A | 28887542 |
| Willer et al(2013) | ebi-a-GCST002216 | Triglycerides | 188577 | N/A | 24097068 |
| Willer et al(2013) | ebi-a-GCST002222 | LDL cholesterol | 188577 | N/A | 24097068 |
| Prins et al(2017) | ebi-a-GCST005068 | LDL cholesterol | 9961 | N/A | 28887542 |
| Willer et al(2013) | ebi-a-GCST002223 | HDL cholesterol | 188577 | N/A | 24097068 |
| Prins et al(2017) | ebi-a-GCST005058 | HDL cholesterol | 9961 | N/A | 28887542 |

**Table S3. IVs' information**

| id.exposure | chr | SNP | effect_allele | other_allele | eaf.exposure | pos.exposure | beta.exposure | pval.exposure | se.exposure | samplesize.exposure |
| --- | --- | --- | --- | --- | --- | --- | --- | --- | --- | --- |
| ebi-a-GCST005038 | 1 | rs2228145 | C | A | 0.648 | 1.54E+08 | 0.0373 | 1.10E-10 | 0.0058 | 360838 |
| ebi-a-GCST005038 | 1 | rs1102705 | A | G | 0.1054 | 1.73E+08 | -0.0591 | 4.87E-09 | 0.0101 | 360838 |
| ebi-a-GCST005038 | 1 | rs1214598 | A | G | 0.3316 | 1.67E+08 | -0.0401 | 2.14E-11 | 0.006 | 360838 |
| ebi-a-GCST005038 | 1 | rs61816766 | C | T | 0.97959 | 1.52E+08 | 0.142 | 6.76E-16 | 0.0176 | 360838 |
| ebi-a-GCST005038 | 1 | rs12123821 | T | C | 0.04762 | 1.52E+08 | 0.1159 | 1.04E-16 | 0.014 | 360838 |
| ebi-a-GCST005038 | 1 | rs4090390 | A | C | 0.2228 | 1.73E+08 | 0.0438 | 5.68E-11 | 0.0067 | 360838 |
| ebi-a-GCST005038 | 1 | rs301802 | A | T | 0.4592 | 8497307 | 0.048 | 1.33E-16 | 0.0058 | 360838 |
| ebi-a-GCST005038 | 1 | rs760805 | T | A | 0.5782 | 25251923 | 0.0374 | 3.79E-10 | 0.006 | 360838 |
| ebi-a-GCST005038 | 1 | rs1289273 | G | A | 0.5731 | 2.27E+08 | 0.035 | 1.09E-09 | 0.0057 | 360838 |
| ebi-a-GCST005038 | 1 | rs4845604 | A | G | 0.1276 | 1.52E+08 | -0.0491 | 5.42E-09 | 0.0084 | 360838 |
| ebi-a-GCST005038 | 2 | rs10174949 | A | G | 0.3163 | 8442248 | -0.0656 | 9.92E-26 | 0.0063 | 360838 |
| ebi-a-GCST005038 | 2 | rs4973380 | A | T | 0.2432 | 2.29E+08 | 0.0397 | 2.59E-09 | 0.0067 | 360838 |
| ebi-a-GCST005038 | 2 | rs10865050 | A | G | 0.1531 | 1.03E+08 | -0.1249 | 6.37E-50 | 0.0084 | 360838 |
| ebi-a-GCST005038 | 2 | rs34290285 | A | G | 0.2653 | 2.43E+08 | -0.0766 | 1.16E-24 | 0.0075 | 360838 |
| ebi-a-GCST005038 | 2 | rs74847330 | G | A | 0.8776 | 1.44E+08 | -0.0497 | 1.67E-08 | 0.0088 | 360838 |
| ebi-a-GCST005038 | 3 | rs73196739 | T | C | 0.1599 | 1.88E+08 | -0.0477 | 7.03E-10 | 0.0077 | 360838 |
| ebi-a-GCST005038 | 3 | rs61192126 | C | T | 0.6905 | 72394852 | -0.0384 | 1.56E-09 | 0.0064 | 360838 |
| ebi-a-GCST005038 | 3 | rs7625643 | G | A | 0.5578 | 1.41E+08 | 0.0346 | 2.96E-09 | 0.0058 | 360838 |
| ebi-a-GCST005038 | 3 | rs9877752 | A | G | 0.4116 | 1.88E+08 | 0.0398 | 4.98E-12 | 0.0058 | 360838 |
| ebi-a-GCST005038 | 3 | rs6800001 | A | G | 0.3418 | 33081102 | 0.033 | 2.28E-08 | 0.0059 | 360838 |
| ebi-a-GCST005038 | 3 | rs75770022 | C | A | 0.9881 | 1.88E+08 | -0.1008 | 1.18E-08 | 0.0177 | 360838 |
| ebi-a-GCST005038 | 3 | rs80064395 | T | C | 0.05782 | 1.96E+08 | -0.0726 | 1.31E-11 | 0.0107 | 360838 |
| ebi-a-GCST005038 | 3 | rs519973 | A | G | 0.3537 | 1.88E+08 | 0.0358 | 3.23E-09 | 0.006 | 360838 |
| ebi-a-GCST005038 | 4 | rs45613035 | C | T | 0.91327 | 1.23E+08 | 0.0602 | 8.12E-10 | 0.0098 | 360838 |
| ebi-a-GCST005038 | 4 | rs10033073 | G | A | 0.6497 | 4775401 | 0.0437 | 1.36E-09 | 0.0072 | 360838 |
| ebi-a-GCST005038 | 4 | rs58939053 | T | C | 0.3435 | 1.23E+08 | 0.0544 | 3.85E-19 | 0.0061 | 360838 |
| ebi-a-GCST005038 | 4 | rs5743618 | A | C | 0.2993 | 38798648 | -0.0915 | 2.73E-42 | 0.0067 | 360838 |
| ebi-a-GCST005038 | 4 | rs228619 | G | A | 0.5306 | 1.04E+08 | 0.0362 | 2.41E-10 | 0.0057 | 360838 |
| ebi-a-GCST005038 | 5 | rs16903574 | G | C | 0.91837 | 14610309 | 0.07 | 1.68E-10 | 0.011 | 360838 |
| ebi-a-GCST005038 | 5 | rs848 | C | A | 0.7636 | 1.32E+08 | -0.0601 | 1.62E-16 | 0.0073 | 360838 |
| ebi-a-GCST005038 | 5 | rs6594499 | A | C | 0.4813 | 1.1E+08 | -0.0723 | 1.10E-36 | 0.0057 | 360838 |
| ebi-a-GCST005038 | 5 | rs35370170 | A | G | 0.04762 | 1.37E+08 | 0.0638 | 8.74E-09 | 0.0111 | 360838 |
| ebi-a-GCST005038 | 5 | rs6881706 | T | G | 0.2925 | 35879156 | -0.0707 | 1.73E-28 | 0.0064 | 360838 |
| ebi-a-GCST005038 | 5 | rs2910162 | G | A | 0.6837 | 1.6E+08 | 0.0344 | 1.54E-08 | 0.0061 | 360838 |
| ebi-a-GCST005038 | 5 | rs740474 | T | C | 0.4167 | 1.41E+08 | -0.0361 | 6.59E-10 | 0.0059 | 360838 |
| ebi-a-GCST005038 | 5 | rs249677 | A | C | 0.3605 | 1.42E+08 | 0.0366 | 7.54E-10 | 0.0059 | 360838 |
| ebi-a-GCST005038 | 5 | rs3853750 | C | T | 0.818 | 1.1E+08 | 0.0848 | 6.51E-27 | 0.0079 | 360838 |
| ebi-a-GCST005038 | 5 | rs3749833 | C | T | 0.7024 | 1.32E+08 | 0.038 | 3.62E-09 | 0.0064 | 360838 |
| ebi-a-GCST005038 | 6 | rs2507978 | A | G | 0.4116 | 31351664 | -0.0407 | 2.51E-11 | 0.0061 | 360838 |
| ebi-a-GCST005038 | 6 | rs2134814 | G | C | 0.6463 | 90987512 | -0.0427 | 1.03E-12 | 0.006 | 360838 |
| ebi-a-GCST005038 | 6 | rs680061 | G | A | 0.6105 | 32571098 | -0.0397 | 1.71E-11 | 0.0059 | 360838 |
| ebi-a-GCST005038 | 6 | rs10947428 | C | T | 0.8061 | 33647058 | 0.0426 | 1.68E-09 | 0.0071 | 360838 |
| ebi-a-GCST005038 | 6 | rs34004019 | G | A | 0.6956 | 32626403 | -0.0913 | 2.52E-38 | 0.0071 | 360838 |
| ebi-a-GCST005038 | 6 | rs41258084 | T | C | 0.03401 | 32780957 | 0.0733 | 5.92E-09 | 0.0126 | 360838 |
| ebi-a-GCST005038 | 6 | rs9372120 | G | T | 0.8248 | 1.07E+08 | 0.0406 | 1.02E-08 | 0.0071 | 360838 |
| ebi-a-GCST005038 | 6 | rs144530872 | A | G | 0.03571 | 29863887 | 0.0998 | 5.71E-11 | 0.0152 | 360838 |
| ebi-a-GCST005038 | 6 | rs2854001 | A | G | 0.1837 | 31323012 | 0.0537 | 3.31E-14 | 0.0071 | 360838 |
| ebi-a-GCST005038 | 6 | rs3128959 | A | G | 0.1241 | 33048380 | -0.0601 | 2.25E-10 | 0.0095 | 360838 |
| ebi-a-GCST005038 | 7 | rs4296977 | T | C | 0.1582 | 77018542 | -0.0534 | 6.92E-11 | 0.0082 | 360838 |
| ebi-a-GCST005038 | 7 | rs4722758 | G | C | 0.7925 | 28156606 | 0.0409 | 6.84E-09 | 0.0071 | 360838 |
| ebi-a-GCST005038 | 7 | rs17664743 | A | G | 0.2041 | 50253897 | 0.041 | 8.27E-09 | 0.0071 | 360838 |
| ebi-a-GCST005038 | 7 | rs6461503 | C | T | 0.5272 | 20560996 | -0.041 | 8.81E-13 | 0.0057 | 360838 |
| ebi-a-GCST005038 | 8 | rs6990534 | G | A | 0.6429 | 1.29E+08 | -0.04 | 1.74E-10 | 0.0063 | 360838 |
| ebi-a-GCST005038 | 8 | rs2221641 | T | C | 0.3878 | 81294702 | -0.0419 | 1.03E-12 | 0.0059 | 360838 |
| ebi-a-GCST005038 | 9 | rs4742127 | C | G | 0.2891 | 5860957 | 0.0348 | 3.94E-08 | 0.0063 | 360838 |
| ebi-a-GCST005038 | 9 | rs12551834 | A | G | 0.07993 | 1.32E+08 | -0.0615 | 2.72E-09 | 0.0103 | 360838 |
| ebi-a-GCST005038 | 9 | rs144829310 | T | G | 0.1565 | 6208030 | 0.0828 | 2.60E-26 | 0.0078 | 360838 |
| ebi-a-GCST005038 | 10 | rs12413578 | T | C | 0.09864 | 9049253 | -0.0934 | 3.31E-23 | 0.0094 | 360838 |
| ebi-a-GCST005038 | 10 | rs4747846 | C | G | 0.4813 | 6074451 | 0.0355 | 1.24E-09 | 0.0059 | 360838 |
| ebi-a-GCST005038 | 10 | rs11255753 | T | G | 0.2568 | 8605553 | 0.0448 | 1.03E-11 | 0.0066 | 360838 |
| ebi-a-GCST005038 | 10 | rs61839672 | A | G | 0.06803 | 6111993 | 0.073 | 6.45E-14 | 0.0097 | 360838 |
| ebi-a-GCST005038 | 10 | rs11256016 | A | G | 0.02381 | 9043352 | 0.0798 | 1.72E-09 | 0.0133 | 360838 |
| ebi-a-GCST005038 | 11 | rs10789841 | C | T | 0.6905 | 1.11E+08 | -0.0456 | 1.48E-12 | 0.0064 | 360838 |
| ebi-a-GCST005038 | 11 | rs12365699 | A | G | 0.1531 | 1.19E+08 | -0.0592 | 3.54E-14 | 0.0078 | 360838 |
| ebi-a-GCST005038 | 11 | rs11033545 | G | T | 0.5578 | 36327582 | 0.0334 | 2.40E-08 | 0.006 | 360838 |
| ebi-a-GCST005038 | 11 | rs479844 | G | A | 0.5714 | 65551957 | 0.0412 | 1.15E-12 | 0.0058 | 360838 |
| ebi-a-GCST005038 | 11 | rs56129466 | G | A | 0.7942 | 1.28E+08 | -0.0495 | 1.25E-12 | 0.007 | 360838 |
| ebi-a-GCST005038 | 11 | rs2212434 | T | C | 0.4286 | 76281593 | 0.0869 | 8.92E-52 | 0.0057 | 360838 |
| ebi-a-GCST005038 | 11 | rs12283565 | T | C | 0.07313 | 76346193 | -0.0733 | 4.77E-12 | 0.0106 | 360838 |
| ebi-a-GCST005038 | 12 | rs6489785 | C | T | 0.631 | 1.21E+08 | -0.0428 | 3.57E-13 | 0.0059 | 360838 |
| ebi-a-GCST005038 | 12 | rs1689510 | C | G | 0.3163 | 56396768 | 0.051 | 3.39E-17 | 0.0061 | 360838 |
| ebi-a-GCST005038 | 12 | rs1059513 | C | T | 0.8895 | 57489709 | -0.0828 | 1.15E-18 | 0.0094 | 360838 |
| ebi-a-GCST005038 | 12 | rs7299924 | G | A | 0.8214 | 50348961 | 0.0416 | 1.94E-08 | 0.0074 | 360838 |
| ebi-a-GCST005038 | 12 | rs73107993 | T | C | 0.2041 | 48195873 | -0.0467 | 1.14E-11 | 0.0069 | 360838 |
| ebi-a-GCST005038 | 13 | rs4943794 | C | G | 0.2262 | 41173408 | 0.0396 | 1.41E-08 | 0.007 | 360838 |
| ebi-a-GCST005038 | 14 | rs1885013 | A | G | 0.3588 | 68754695 | -0.0382 | 1.33E-09 | 0.0063 | 360838 |
| ebi-a-GCST005038 | 15 | rs56375023 | A | G | 0.2126 | 67448363 | 0.0731 | 3.11E-27 | 0.0068 | 360838 |
| ebi-a-GCST005038 | 15 | rs10519067 | A | G | 0.1395 | 61068347 | -0.0518 | 1.10E-09 | 0.0085 | 360838 |
| ebi-a-GCST005038 | 15 | rs12440045 | C | A | 0.5476 | 41782684 | 0.0403 | 7.68E-12 | 0.0059 | 360838 |
| ebi-a-GCST005038 | 15 | rs8030821 | T | A | 0.6105 | 70563984 | 0.0337 | 1.54E-08 | 0.006 | 360838 |
| ebi-a-GCST005038 | 15 | rs3540 | A | G | 0.3452 | 91045408 | -0.0346 | 1.28E-08 | 0.0061 | 360838 |
| ebi-a-GCST005038 | 16 | rs2241099 | G | C | 0.7381 | 11225064 | -0.0697 | 4.62E-26 | 0.0066 | 360838 |
| ebi-a-GCST005038 | 16 | rs3024665 | T | C | 0.07653 | 27371539 | 0.0665 | 2.18E-08 | 0.0119 | 360838 |
| ebi-a-GCST005038 | 17 | rs12941864 | T | C | 0.466 | 1395682 | -0.0335 | 4.98E-08 | 0.0061 | 360838 |
| ebi-a-GCST005038 | 17 | rs11658582 | G | C | 0.5663 | 38763200 | 0.0563 | 2.07E-21 | 0.0059 | 360838 |
| ebi-a-GCST005038 | 17 | rs34666276 | C | T | 0.648 | 47379486 | 0.0397 | 2.31E-11 | 0.0059 | 360838 |
| ebi-a-GCST005038 | 17 | rs146346285 | T | C | 0.02721 | 38897220 | 0.1541 | 1.59E-08 | 0.0273 | 360838 |
| ebi-a-GCST005038 | 17 | rs7224129 | A | G | 0.4728 | 38075426 | 0.0535 | 8.11E-21 | 0.0057 | 360838 |
| ebi-a-GCST005038 | 19 | rs10414065 | T | C | 0.07823 | 33721455 | -0.0917 | 2.29E-14 | 0.012 | 360838 |
| ebi-a-GCST005038 | 20 | rs6021264 | G | A | 0.92347 | 50134683 | -0.0636 | 1.04E-08 | 0.0111 | 360838 |
| ebi-a-GCST005038 | 20 | rs6011033 | G | A | 0.7755 | 62322699 | 0.0437 | 2.66E-10 | 0.0069 | 360838 |
| ebi-a-GCST005038 | 20 | rs2766678 | A | G | 0.2126 | 52208356 | -0.0559 | 2.95E-14 | 0.0074 | 360838 |
| ebi-a-GCST005038 | 22 | rs5758343 | T | A | 0.7823 | 41816652 | -0.0474 | 2.17E-11 | 0.0071 | 360838 |
| ebi-a-GCST006862 | 2 | rs3771180 | T | G | NA | 1.03E+08 | -0.17425 | 1.47E-20 | 0.018746 | 127669 |
| ebi-a-GCST006862 | 5 | rs6893213 | T | C | NA | 1.1E+08 | 0.145249 | 7.99E-11 | 0.022344 | 127669 |
| ebi-a-GCST006862 | 5 | rs6894249 | G | A | NA | 1.32E+08 | 0.087396 | 2.15E-11 | 0.013053 | 127669 |
| ebi-a-GCST006862 | 5 | rs7705042 | A | C | NA | 1.41E+08 | 0.079566 | 8.53E-10 | 0.01297 | 127669 |
| ebi-a-GCST006862 | 5 | rs10455025 | C | A | NA | 1.1E+08 | 0.140203 | 2.03E-25 | 0.013456 | 127669 |
| ebi-a-GCST006862 | 5 | rs20541 | G | A | NA | 1.32E+08 | -0.11964 | 1.36E-14 | 0.015538 | 127669 |
| ebi-a-GCST006862 | 6 | rs2596464 | C | T | NA | 31412961 | 0.093668 | 1.79E-13 | 0.01272 | 127669 |
| ebi-a-GCST006862 | 6 | rs2325291 | A | G | NA | 90986686 | -0.09554 | 8.58E-13 | 0.01336 | 127669 |
| ebi-a-GCST006862 | 6 | rs2523716 | T | C | NA | 30170525 | 0.089234 | 1.38E-08 | 0.015721 | 127669 |
| ebi-a-GCST006862 | 6 | rs241429 | G | A | NA | 32803840 | -0.07233 | 2.98E-08 | 0.013051 | 127669 |
| ebi-a-GCST006862 | 6 | rs9272346 | A | G | NA | 32604372 | 0.147072 | 2.35E-28 | 0.013317 | 127669 |
| ebi-a-GCST006862 | 6 | rs2855812 | T | G | NA | 31472720 | 0.093118 | 2.80E-10 | 0.014759 | 127669 |
| ebi-a-GCST006862 | 6 | rs1233578 | G | A | NA | 28712247 | 0.104861 | 5.32E-09 | 0.017965 | 127669 |
| ebi-a-GCST006862 | 8 | rs10957979 | G | A | NA | 81289787 | -0.07392 | 2.33E-08 | 0.013234 | 127669 |
| ebi-a-GCST006862 | 9 | rs992969 | G | A | NA | 6209697 | -0.15815 | 4.27E-29 | 0.014126 | 127669 |
| ebi-a-GCST006862 | 10 | rs1663687 | A | G | NA | 9054787 | -0.08431 | 1.52E-10 | 0.013167 | 127669 |
| ebi-a-GCST006862 | 11 | rs2155219 | T | G | NA | 76299194 | 0.105003 | 2.90E-15 | 0.013299 | 127669 |
| ebi-a-GCST006862 | 12 | rs167769 | T | C | NA | 57503775 | 0.075948 | 5.50E-09 | 0.013024 | 127669 |
| ebi-a-GCST006862 | 15 | rs10519067 | A | G | NA | 61068347 | -0.12082 | 1.49E-10 | 0.018861 | 127669 |
| ebi-a-GCST006862 | 15 | rs17293632 | T | C | NA | 67442596 | 0.117968 | 8.81E-16 | 0.014668 | 127669 |
| ebi-a-GCST006862 | 16 | rs12935657 | A | G | NA | 11219041 | -0.10374 | 2.06E-12 | 0.014756 | 127669 |
| ebi-a-GCST006862 | 17 | rs17637472 | A | G | NA | 47461433 | 0.077675 | 3.28E-09 | 0.013127 | 127669 |
| ebi-a-GCST006862 | 17 | rs2305479 | T | C | NA | 38062217 | -0.17822 | 1.00E-42 | 0.013008 | 127669 |
| ieu-a-996 | 1 | rs2477121 | T | A | 0.571863 | 1.5E+08 | 0.099648 | 1.45E-08 | 0.017571 | 40529 |
| ieu-a-996 | 1 | rs12144049 | T | C | 0.677644 | 1.52E+08 | -0.20181 | 2.80E-27 | 0.018641 | 40530 |
| ieu-a-996 | 1 | rs61815704 | G | C | 0.023431 | 1.53E+08 | 0.54253 | 3.91E-20 | 0.05899 | 34563 |
| ieu-a-996 | 2 | rs6419573 | C | T | 0.708531 | 1.03E+08 | -0.12395 | 2.92E-10 | 0.01965 | 40529 |
| ieu-a-996 | 5 | rs12188917 | C | T | 0.205033 | 1.32E+08 | 0.170064 | 2.89E-15 | 0.021524 | 40530 |
| ieu-a-996 | 6 | rs4151657 | C | T | 0.33741 | 31917540 | 0.10198 | 7.86E-09 | 0.017657 | 40531 |
| ieu-a-996 | 8 | rs12334935 | A | G | 0.473769 | 1.27E+08 | 0.092611 | 4.18E-08 | 0.016878 | 40529 |
| ieu-a-996 | 11 | rs479844 | G | A | 0.548407 | 65551957 | 0.143746 | 3.45E-17 | 0.017038 | 40531 |
| ieu-a-996 | 11 | rs10790275 | C | G | 0.7512 | 1.19E+08 | 0.122434 | 2.16E-08 | 0.021853 | 40834 |
| ieu-a-996 | 11 | rs2212434 | T | C | 0.450704 | 76281593 | 0.129133 | 2.09E-14 | 0.016879 | 40530 |
| ieu-a-996 | 17 | rs8066625 | A | G | 0.107212 | 40390629 | 0.175585 | 3.84E-08 | 0.031912 | 40529 |
| ieu-a-996 | 19 | rs2918299 | T | C | 0.166014 | 8787273 | 0.142564 | 5.45E-10 | 0.022955 | 38477 |
| ieu-a-996 | 20 | rs6062486 | A | G | 0.647639 | 62302539 | 0.104574 | 2.40E-08 | 0.018726 | 40531 |

**Table S4. MR of TC**

| Exposure | ID | Outcome | ID | SNP number | Methods | β | SE | P | P of pleiotropy test | P of reverse MR |
| --- | --- | --- | --- | --- | --- | --- | --- | --- | --- | --- |
| Allergic disease (asthma, hay fever or eczema) | ebi-a-GCST005038 | Cholesterol, total | ebi-a-GCST002221 | 30 | Inverse variance weighted | -0.06589 | 0.02689 | 0.016534 | 0.644349 | 0.152 |
| Allergic disease (asthma, hay fever or eczema) | ebi-a-GCST005038 | Cholesterol, total | ebi-a-GCST005065 | 66 | Inverse variance weighted | -0.04255 | 0.042615 | 0.318053 | 0.094727 | 0.192 |
| Asthma | ebi-a-GCST006862 | Cholesterol, total | ebi-a-GCST002221 | 23 | Inverse variance weighted | -0.05734 | 0.015949 | 0.000324 | 0.710094 | 0.437 |
| Asthma | ebi-a-GCST006862 | Cholesterol, total | ebi-a-GCST005065 | 19 | Inverse variance weighted | -0.05391 | 0.041798 | 0.197132 | 0.448642 | 0.273 |
| Eczema | ieu-a-996 | Cholesterol, total | ebi-a-GCST002221 | 4 | Inverse variance weighted | -0.00809 | 0.022086 | 0.714167 | 0.238515 | 0.899 |
| Eczema | ieu-a-996 | Cholesterol, total | ebi-a-GCST005065 | 9 | Inverse variance weighted | 0.009915 | 0.039188 | 0.80025 | 0.954105 | 0.821 |

**Table S5. MR of TG**

| Exposure | ID | Outcome | ID | SNP number | Methods | β | SE | P | P of pleiotropy test | P of reverse MR |
| --- | --- | --- | --- | --- | --- | --- | --- | --- | --- | --- |
| Allergic disease (asthma, hay fever or eczema) | ebi-a-GCST005038 | Triglycerides | ebi-a-GCST002216 | 28 | Inverse variance weighted | 0.025131 | 0.020587 | 0.222201 | 0.114533 | 0.054 |
| Asthma | ebi-a-GCST006862 | Triglycerides | ebi-a-GCST002216 | 23 | Inverse variance weighted | -0.01672 | 0.017193 | 0.330743 | 0.40548 | 0.076 |
| Eczema | ieu-a-996 | Triglycerides | ebi-a-GCST002216 | 4 | Inverse variance weighted | 0.020672 | 0.020835 | 0.321121 | 0.844125 | 0.880 |

**Table S6. MR of LDL**

| Exposure | ID | Outcome | ID | SNP number | Methods | β | SE | P | P of pleiotropy test | P of reverse MR |
| --- | --- | --- | --- | --- | --- | --- | --- | --- | --- | --- |
| Allergic disease (asthma, hay fever or eczema) | ebi-a-GCST005038 | LDL cholesterol | ebi-a-GCST002222 | 30 | Inverse variance weighted | -0.05122 | 0.027923 | 0.066603 | 0.863046 | 0.457 |
| Allergic disease (asthma, hay fever or eczema) | ebi-a-GCST005038 | LDL cholesterol | ebi-a-GCST005068 | 59 | Inverse variance weighted | -0.03759 | 0.047744 | 0.431152 | 0.095732 | 0.645 |
| Asthma | ebi-a-GCST006862 | LDL cholesterol | ebi-a-GCST002222 | 23 | Inverse variance weighted | -0.03653 | 0.013789 | 0.008077 | 0.542639 | 0.679 |
| Asthma | ebi-a-GCST006862 | LDL cholesterol | ebi-a-GCST005068 | 20 | Inverse variance weighted | -0.02212 | 0.037384 | 0.554027 | 0.178303 | 0.456 |
| Eczema | ieu-a-996 | LDL cholesterol | ebi-a-GCST002222 | 4 | Inverse variance weighted | -0.03598 | 0.028597 | 0.208385 | 0.242931 | 0.101 |
| Eczema | ieu-a-996 | LDL cholesterol | ebi-a-GCST005068 | 7 | Inverse variance weighted | -0.01046 | 0.04684 | 0.823289 | 0.335266 | 0.493 |

**Table S7. MR of HDL**

| Exposure | ID | Outcome | ID | SNP number | Methods | β | SE | P | P of pleiotropy test | P of reverse MR |
| --- | --- | --- | --- | --- | --- | --- | --- | --- | --- | --- |
| Allergic disease (asthma, hay fever or eczema) | ebi-a-GCST005038 | HDL cholesterol | ebi-a-GCST002223 | 28 | Inverse variance weighted | -0.02525 | 0.032004 | 0.430051 | 0.967193 | 0.544 |
| Allergic disease (asthma, hay fever or eczema) | ebi-a-GCST005038 | HDL cholesterol | ebi-a-GCST005058 | 64 | Inverse variance weighted | 0.08638 | 0.055757 | 0.121328 | 0.788792 | 0.746 |
| Asthma | ebi-a-GCST006862 | HDL cholesterol | ebi-a-GCST002223 | 23 | Inverse variance weighted | -0.03714 | 0.017111 | 0.029972 | 0.575226 | 0.183 |
| Asthma | ebi-a-GCST006862 | HDL cholesterol | ebi-a-GCST005058 | 17 | Inverse variance weighted | 0.009891 | 0.042277 | 0.81501 | 0.518781 | 0.850 |
| Eczema | ieu-a-996 | HDL cholesterol | ebi-a-GCST002223 | 4 | Inverse variance weighted | 0.018589 | 0.017075 | 0.276316 | 0.328088 | 0.186 |
| Eczema | ieu-a-996 | HDL cholesterol | ebi-a-GCST005058 | 7 | Inverse variance weighted | 0.011483 | 0.072535 | 0.874213 | 0.281278 | 0.751 |
